# Effect of the Presence of Stem on Quality of Oolong Tea

**DOI:** 10.3390/foods11213439

**Published:** 2022-10-29

**Authors:** Jiazheng Lin, Yuwan Wang, Lin Chen, Yunfei Yang, Zheng Tu, Yang Ye

**Affiliations:** 1 Tea Research Institute, Chinese Academy of Agricultural Sciences, Hangzhou 310008, China; 2 Department of Tea Science, Zhejiang University, Hangzhou 310058, China

**Keywords:** stems, oolong tea, taste components, free amino acids

## Abstract

Combined with the unique processing technology of oolong tea, oolong tea with stem processing has a better flavor compared to oolong tea without stem processing. However, there is currently no available evidence to support the contribution of stems to the taste quality of oolong tea. In this study, the electronic tongue, sensory evaluation method combined with liquid chromatography, and gas chromatography–mass spectrometry were used to explore the influence of the presence of stems on the flavor substances and aroma of oolong tea during processing. The results showed that the presence of stems significantly increased the umami taste of oolong tea (*p* < 0.05), and the content of seven free amino acids (*p* < 0.05), including theanine (53.165 μg/mL) and aspartic acid (3.190 μg/mL), two umami-related amino acids, significantly increased. Moreover, the content of nerolidol (2.598 μg/g) in aroma components was significantly increased. This study identifies the contribution of stems to oolong tea quality during processing.

## 1. Introduction

Tea is an ideal beverage that is widely consumed around the world. According to the degree of fermentation, it can be divided into unfermented tea, semifermented tea, and fermented tea [1]. Oolong tea is a typical semifermented tea, and its unique flavor profile means it is widely consumed in China, Japan, and other regions [2]. Studies have shown that the quality components that affect the taste of oolong tea include catechin (bitter) [3], caffeine (bitter) [4], free amino acids (umami and sweet) [5], soluble sugar (sweet) [6], flavonoids (astringent) [7], and theaflavins (astringent) [8], etc. The mutual transformation and formation of these components during the processing of oolong tea lay the material basis for its taste [9].

Oolong tea processing includes several production processes such as withering, shaking, firing, rolling, and drying. Studies have shown that catechin content gradually decreases during oolong tea processing [10], and free amino acid and soluble sugar contents generally increase. The aroma characteristics of tea products made from different oolong tea varieties show a unique “variety fragrance” [11]. More importantly, in the processing of oolong tea, the limited moisture, and free amino acids, catechins, and other quality components in the fresh leaves and stems can be transferred between the stems and leaves many times and gradually utilized, commonly known as “zou shui huan yang” [12]. Different from other tea processing methods, oolong tea is processed with fresh leaves and stems, and finally, the stems are removed to obtain tea products. It is traditionally believed that stems contain high water content, and various substances are dissolved in water. As the water in the leaves evaporates, the quality components are transported to the leaves, enriching and enhancing the taste of oolong tea, but this concept has not been confirmed. In recent years, Zeng et al. showed that the presence of stems could not significantly improve the aroma of oolong tea [13]. They found that the content of free amino acids (especially theanine) in oolong tea made from leaves combined with stems was significantly higher than that of tea only made from leaves. However, whether the change in free amino acid content is due to stem transport or enhanced auto metabolism is unknown.

Currently, the quality of tea can be evaluated by the electronic tongue, liquid chromatography–mass spectrometry, and gas chromatography–mass spectrometer (GC-MS). Among them, the electronic tongue can not only quickly imitate the sense of human taste but can also express taste more sensitively, automatically, and fairly compared to human tasters. It can also be used to distinguish different grades and different origins of tea. Yan et al. effectively judged the geographical origin of Anji white tea through the electronic tongue, seven independent sensors, chemometrics, and other methods [14]. Chen et al. used UPLC-Q-TOF/MS and GC-TOF/MS on oolong tea throughout the processing process and observed that catechins and oxidation products, flavonol glycosides, and amino acids were the key identifying metabolites [15]. Zeng et al. obtained (*E*, *E*)-2,4-Heptadienal, (*E*)-2-octenal, benzeneacetaldehyde, and (*E*)-nerolidol as the key volatile compounds to distinguish different grades of Tieguanyin by HS-SPME-GC-MS technique [16].

The presence of the stem may have a positive effect on the taste and aroma quality of oolong tea. This study was based on the production process of clear aroma oolong tea, mainly through the whole shoot (combined leaf and combined stem), separated stem, and separated leaf—three tea raw material harvesting parts for the processing of oolong tea. The overall evaluation of the taste quality of tea leaves was carried out by electronic tongue and sensory evaluation methods. The taste and aroma components of oolong tea were analyzed by high-performance liquid chromatography (HPLC), ultra-performance liquid chromatography (UPLC), UV-2550 spectrometer, and GC-MS, and the presence of transfer of amino acid fractions through the stem during the processing of oolong tea was investigated to explore the actual role of the stem in the processing of oolong tea from the perspective of taste and aroma.

## 2. Materials and Methods

### 2.1. Chemicals and Reagents

Catechin (C, ≥98%), gallocatechin (GC, ≥98%), gallocatechin-3-gallate (GCG, ≥98%), epicatechin (EC, ≥98%), epicatechin-3-gallate (ECG, ≥98%), epigallocatechin (EGC, ≥98%), epigallocatechin-3-gallate (EGCG, ≥98%), caffeine (CAF, ≥98%), theaflavins (TF, ≥95%), theaflavins-3-gallate (TF3G, ≥98%), theaflavin-3′-gallate (TF3′G, ≥98%), theaflavine-3,3′-digallate (TFDG, ≥98%), quercetin-3-O-galactoside (≥98%), myricetin-3-O-galactoside (≥98%), kaempferol-3-O-rutinoside (≥98%), vitexin (≥98%), astragaloside (≥98%), quercetin (≥98%), and kaempferol (≥98%) were purchased from Shanghai yuanye Bio-Technology Co., Ltd. (Shanghai, China). l-alanine, l-arginine, l-aspartic acid, l-cystine, l-glutamic acid, glycine, l-histidine, l-isoleucine, l-leucine, l-lysine, l-methionine, l-phenylalanine, l-proline, l-serine, l-threonine, l-tyrosine, l-valine, l-asparagine, l-glutamine, theanine, γ-aminobutyric acid, and l-tryptophan were purchased from Sigma (Sigma-Aldrich Co., St. Louis, MO, USA). Anthrone reagent and ninhydrin/formic acid reducing agent were purchased from BeiJing DingGuochangSheng Biotech. Co., Ltd. (Bejing, China). Ethyl caprate (≥99%) was purchased from Shanghai Macklin Biochemical Technology Co., Ltd. (Shanghai, China), and n-alkane mixed standard C7-C40 was purchased from o2si (o2si smart solutions—an LGC Standards Company, Charleston, SC, USA).

### 2.2. Oolong Tea Processing and Sample Preparation

**Figure 1:**
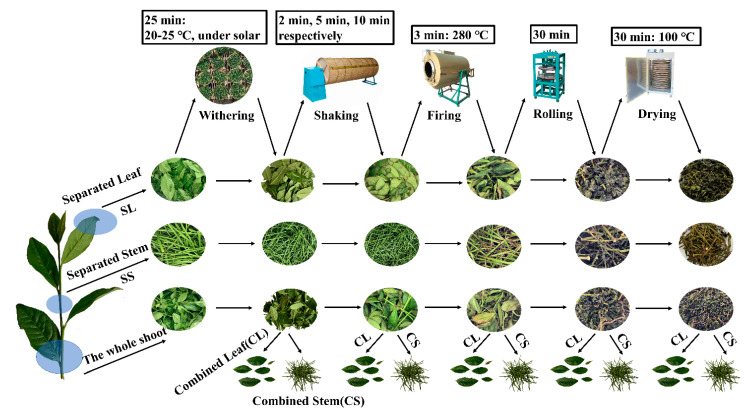
Manufacturing processes of oolong tea.

The raw materials of fresh leaves were Jin Guanyin variety, which was picked in the tea plantation at the Shengzhou site of the Tea Research Institute of the Chinese Academy of Agricultural Sciences (29°35′ N, 120°49′ E), Shengzhou, Zhejiang, China, in May 2020. The samples were divided into two categories. The first category was the combined stem (CS) and the combined leaf (CL), which were separated into the combined leaf group and combined stem group after the fresh leaves were picked and processed in various steps. The second category was the stem (separated stem, SS) and leaf (separated leaf, SL), which were directly separated from fresh leaves after they were picked. The processing steps are shown in Figure 1. First, the collected fresh leaves were separated from stems and leaves, and then divided into tea leaves, SL and SS, withered in the sun for 30 min, placed in an air-conditioned room at 18 °C for 30 min, and then shaken three times (respectively 2 min, 5 min, 10 min of shaking). At the end of each shake, the tea leaves were taken out and placed in an air-conditioned room at 18 °C for 90 min. Next, the tea samples were placed in a drum at 280 °C for 5 min to block the enzymatic activity. Finally, the tea was wrapped and kneaded for 20 min, and dried at 100 °C for 25 min. The dried SL and CL were the finished oolong tea. At the end of each processing stage, the tea leaves were separated from stems and leaves (CL and CS), and the SL and SS samples were separately sampled and stored in liquid nitrogen (−80 °C) for further analysis. The process was repeated three times for three sets of samples.

### 2.3. Electronic Tongue Analysis

The AS-TREE electronic tongue system developed by Alpha M.O.S in France was adopted. In this system, five sensors (AHS, CTS, NMS, AND, and SCS) correspond to the five taste factors of sourness, saltiness, umami, sweetness, and bitterness, respectively. For electronic tongue determination at room temperature, 3 g CL and SL finished oolong tea samples were brewed in a 150 mL review cup for 4 min, and 80 mL tea infusion samples were placed in a special beaker (150 mL). This was repeated 3 times, with the acquisition time of 120 s, with data collected every second, and the stable data obtained between 100 and 120 s were used as the output value.

### 2.4. Sensory Evaluation

Refer to Zeng et al. ’s method and modify it slightly [13]. Five senior tea reviewers (2 women and 3 men) and 15 regular consumers (8 women and 7 men) were selected to form a group to participate in the preference score of oolong tea infusion. Since the oolong tea processing involved in the experiment was by Chinese tea production standards, it was nontoxic and nonhazardous. Therefore, this experiment did not require ethical approval. All respondents had to sign an informed consent form before the test, which outlined the purpose, time, and program of the evaluation and the relevant risks to be undertaken by the respondents. A representative tea sample (3.0 g) was then placed in an evaluation cup, filled with boiling water (150 mL), covered with a lid, and brewed for 5 min. All respondents rated the CL and SL oolong tea infusions on a scale from “very dislike” (1 point) to “very much like” (5 points). The temperature of the tea at evaluation or consumption was 65–70 °C.

### 2.5. Analysis of Taste Components

All samples were vacuum-freeze-dried and ground into powder before detection. The lyophilization procedure for vacuum freeze drying was −40 °C for 7 h, −30 °C for 2 h, −20 °C for 2 h, −10 °C for 2 h, −5 °C for 2 h, 0 °C for 7 h, 5 °C for 10 h, 10 °C for 10 h, 15 °C for 10 h, 20 °C for 10 h, and 25 °C for 10 h. The powder samples (3 g/150 mL) were leached in a boiling water bath for 45 min, diluted with distilled water, and fixed to volume for subsequent chemical composition detection.

#### 2.5.1. Analysis of Catechins, Caffeine, and Theaflavins

The contents of catechin, caffeine, and theaflavin were determined simultaneously by ultra-performance liquid chromatography (Ultra Performance Liquid Chromatography, UPLC, Waters, DE, USA). The finished oolong tea samples of CL and SL were taken for detection. The sample solution was filtered through a 0.22 μm membrane, and a mobile phase consisting of 0.1% formic acid aqueous solution (A) and pure acetonitrile (B) was used for chromatographic separation. The column was Waters BEH C18 (1.7 um, 2.1 × 100 mm, Waters, USA), the bath temperature was kept at 15 °C, and the column temperature was kept at 30 °C. The flow rate and injection volume were 350 uL/min and 2 μL, respectively. The gradient settings were as follows: 0–3 min, 60% B; 3–6 min, 90–83% B; 6–12 min, 83–73% B; 12–15 min, 73–50% B; 15–15.5 min, 50% B; 15.5–16 min, 50–90% B; 16–20 min, 90% B.

#### 2.5.2. Analysis of Free Amino Acids

The content of each free amino acid was determined using an amino acid analyzer (Sykam, Eresing, Germany) [17]. All sample solutions were filtered through a 0.45 μm membrane and injected into an amino acid analyzer with a Physiological Li C4 system coupled to an S4300 post-column derivatization system. The separation column was a high-efficiency sodium cation exchange column (4.0 × 150 mm; Pickering Laboratories, Inc., CA, USA). The Sykam S433D Physiological Li C4 system operates with a mobile phase consisting of lithium citrate at pH 2.9, pH 4.2, and pH 8.0, with UV-VIS detection at 570 nm and 440 nm. The flow rate of the mobile phase was 0.45 mL/min, and the flow rate of the derivatizing reagent (ninhydrin) was 0.25 mL/min. The column temperature was 38 °C, and the post-column reaction equipment was maintained at 130 °C. The temperature of the autosampler was maintained at 5 °C, and the injection volume of the standard and samples was 50 μL.

#### 2.5.3. Analysis of Flavonoids and Flavonoid Glycosides

Flavonoids and flavonoid glycosides were detected by high-performance liquid chromatography (HPLC, Shimadzu, Japan) [18]. The finished oolong tea samples of CL and SL were taken for detection, and the sample solution was first filtered through a 0.45 μm membrane. The chromatographic conditions were as follows: DiamonsilTM C18 (4.6 mm × 250 mm) column, particle size 5 μm; mobile phase A was acetic acid/water (0.1:99.9, *v*/*v*); mobile phase B was acetonitrile; the flow rate was 1 mL/min; the column temperature was 35 °C; the UV detection wavelength was 360 nm; the injection volume was 10 μL; gradient elution time was 0–5 min, 3–16.5% B; 5–18 min, 16.5–20% B; 18–23 min, 20–21.2% B; 23–28 min, 21.2% B; 28–38 min, 21.2–30% B; 38–50 min, 30% B; 50–55 min, 30–3% B; 55–60 min, 3% B.

#### 2.5.4. Soluble Sugar Analysis

Total sugar was detected by anthrone–sulfuric acid reaction using a UV-2550 spectrophotometer (Shimadzu, Japan), and glucose was used as a standard [19]. The finished oolong tea samples of CL and SL were taken for detection, and 2 mL of sample solution was reacted with 8 mL of anthrone reagent (0.2 g of anthrone was dissolved in 100 mL of analytically pure sulfuric acid) at 100 °C for 10 min, and then cooled for 10 min. The absorbance was measured at 620 nm from minimum temperature to room temperature, and distilled water was used as a blank sample. The linear equation of standard glucose was C_(mg/mL)_ = (168.48 × A_620_) − 8.7016 (R^2^ = 0.993). C stands for glucose concentration.

### 2.6. Determination of Moisture Content

The moisture content of the tea samples was determined by the gravimetric method. The empty crucible was dried in an oven at 105 °C for 1 h and cooled in a desiccator and weighed (W1). A 5 g sample was placed in a crucible (W2) and dried in a 105 °C oven, cooled in a desiccator, and the drying was repeated until a constant weight (W3) was reached. After that, the moisture content of the tea leaves was calculated according to the following formula: moisture content = (W3 − W1)/(W2 − W1) × 100%

### 2.7. Aroma Component Analysis

Extraction of aroma components: 0.1 g of finished oolong tea samples of the CL group and SL group were weighed and placed into a 12 mL headspace bottle, and 5 mL of boiling ultrapure water and 10 μL of ethyl caprate (internal standard, 10 mg/L) were quickly added. The cap was equilibrated for 5 min, and then the PDMS extraction head was inserted into the headspace vial. After extraction and adsorption for 60 min under the condition of a constant temperature water bath at 60 °C, the solution was immediately resolved at the 250 °C injection port of GC-MS for 5 min.

GC-MS (Agilent, Santa Clara, CA, USA) conditions: column: DB-5MS (Agilent, USA) quartz capillary column (30 m × 0.25 mm × 0.25 μm); oven temperature: 40 °C; inlet temperature: 250 °C; split ratio: 15:1; pressure 48.745 kPa; column flow: 1 mL/min; injection carrier gas: He (99.9999%). Heating program: hold at 40 °C for 2 min, increase to 85 °C at 2 °C/min, hold for 2 min, then increase to 180 °C at 2.5 °C/min, hold for 2 min, then increase to 230 °C at 10 °C/min, hold for 2 min. Electron ionization source: electron energy: 70 eV; ion source temperature: 230 °C; full scan; mass scan range: *m*/*z* 40–400.

Qualitative and quantitative analysis: the NIST 14 spectral library was used to search the volatile components detected by GC-MS, and the retention index of the component was calculated based on the retention time of each component and its adjacent n-alkane retention time. The retention index was qualitatively compared with the retention index in the literature. The internal standard method was used to quantitatively analyze each compound, that is, according to the ratio of the chromatographic peak area of each component to the peak area of the internal standard ethyl caprate.

### 2.8. Statistical Analysis

A Student’s *t*-test and two-way ANOVA were performed using GraphPad Prism 5.01 (GraphPad Prism Inc., San Diego, CA, USA). Data represent the mean (*n* = 3), and significant differences are indicated as * (*p* < 0.05) and ** (*p* < 0.01). The radar chart was performed using Excel (Microsoft Corp., Redmond, WA, USA). Principal component analysis (PCA), correlation analysis, and heat map hierarchical cluster analysis were performed using R package factoextra, psych, and pheatmap, separately. The network was performed using the R package igraph and Cytoscape 3.72 (accessed on 6 June 2022 at http://www.cytoscape.org/).

## 3. Results and Discussion

### 3.1. Comparison of Taste Components

**Figure 2:**
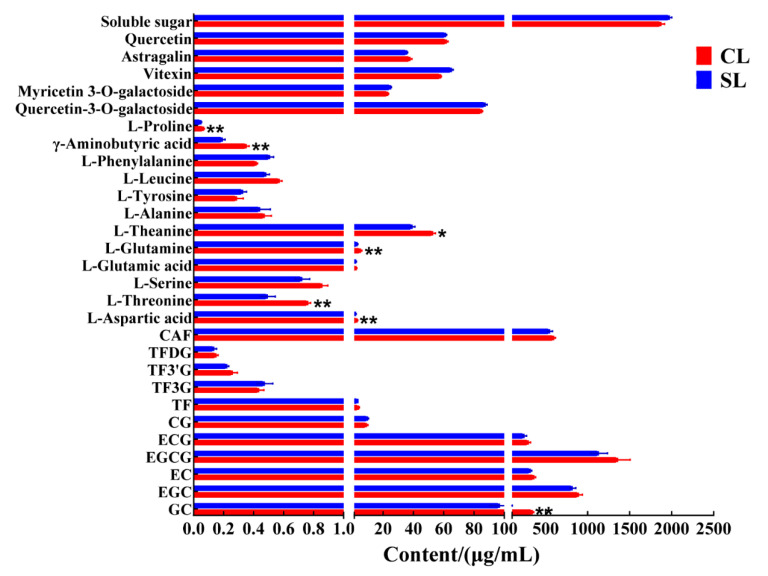
Contents of taste-related components in oolong tea infusion (CL, combined leaf; CS, combined stem; **, *p* < 0.01; *, *p* < 0.05).

**Figure 3:**
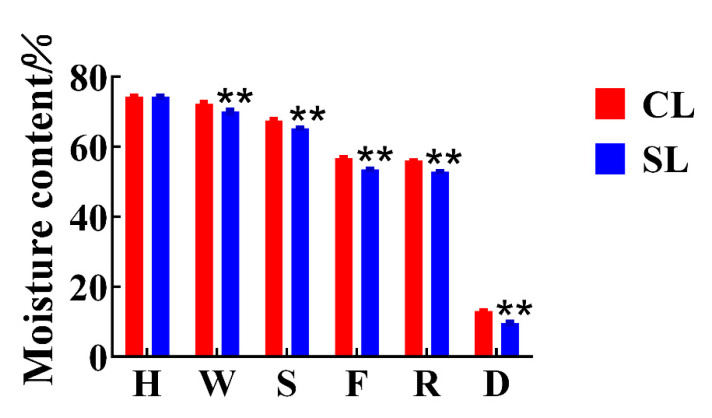
Changes in moisture content during oolong tea processing. H stands for harvesting, W stands for withering, S stands for shaking, F stands for firing, R stands for rolling, and D stands for drying (CL, combined leaf; SL, separated leaf; **, *p* < 0.01).

We detected and analyzed the taste-related components of the finished oolong tea infusions of CL and SL by UPLC and HPLC. The results of the analysis showed that CL had higher levels of many compounds than SL did (Figure 2). The catechins in tea, especially the ester catechins, have always been considered the main substances affecting the bitterness of tea infusion [3,20]. However, we found no significant difference in ester catechins between the two treatments by Student’s *t*-test. In addition, other related chemical components that affect the bitterness of tea, such as caffeine, main flavonoids, and theaflavins, also showed no significant difference. On the other hand, there was no significant difference in the detection of total sugar. The content of free amino acids is positively correlated with the taste of oolong tea, which is the basis for determining the taste and price of oolong tea [21]. So far, 26 amino acids have been identified in tea, including 20 protein amino acids and 6 non-protein amino acids [12]. The contribution of amino acids to taste characteristics can be divided into umami, sweet, bitter, and tasteless [22]. Theanine, aspartic acid, and glutamic acid are the main umami amino acids; bitter amino acids mainly refer to histidine, valine, and tryptophan; glycine, alanine, glutamine, threonine, and serine are sweet amino acids [23]. We detected a total of 12 free amino acids from the finished tea. The results showed that the content of six free amino acids in oolong tea in CL was significantly higher than that in SL, including aspartic acid, threonine, glutamine, theanine, γ-aminobutyric acid, and proline. Most of these free amino acids help to improve the umami and sweetness of oolong tea. The main flavor components of the two groups of oolong tea had the largest difference in the content of free amino acids, that is, the presence of stems during processing promoted the retention of most of the free amino acids in leaves, which is consistent with previous research results [13]. During the processing of oolong tea, the inclusions are transported from the stem to the leaves along with the moisture [12], and we also found that the moisture content of CL was significantly higher than that of SL before drying (Figure 3).

### 3.2. Flavor Quality Analysis

**Figure 4:**
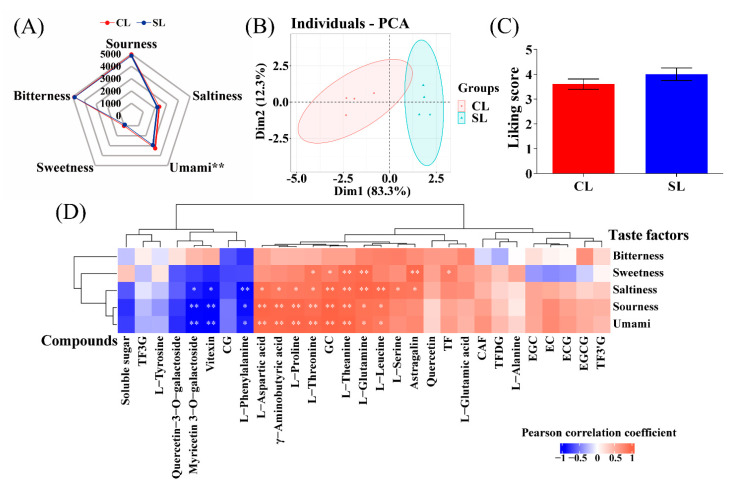
Analysis of the taste of oolong tea. (**A**) Radar map of electronic tongue dataset. (**B**) Principal component analysis (PCA) score chart of taste factor response value. (**C**) Sensory evaluation. (**D**) Heatmap of the correlation between the content of taste components and the response values of taste factors of the electronic tongue (CL, combined leaf; SL, separated leaf; **, *p* < 0.01; *, *p* < 0.05).

The taste factor intensity of the tea infusion in finished oolong tea was evaluated by the electronic tongue to show the influence of the presence of stems on the taste of the finished tea (Figure 4A). It can be seen that the intensity of each taste factor in CL was higher than that in SL, and the umami intensity reached a significant level (*p* < 0.05). It is shown that oolong tea processed with stems was more umami compared to oolong tea processed from leaves only. The above-mentioned taste intensity factors were subjected to PCA by the R package factoextra, which classified all samples according to the response value of each taste factor. The results showed that the tea taste phenotypes of the two processing methods were well differentiated. The contribution rate of principal component 1 (PC1) was 81.3%, and the contribution rate of principal component 2 (PC2) was 18.3%. The cumulative contribution rate reached 99.6% (Figure 4B), indicating that combined stem processing had a significant impact on the overall taste of oolong tea infusion. On the other hand, the average scores (*n* = 20) of the 20 panel members’ preference for the two oolong tea infusions are shown in Figure 4C. The score of SL was higher than that of CL, but the difference was not significant, indicating that the presence of stems did not significantly affect the participants’ preference for the overall characteristics of oolong tea products.

To explore the reasons for the differences in taste factors between the two treatments from the perspective of compounds, we used the R package psych to calculate the correlations of 29 compounds in CL and SL oolong tea with five flavor factors. Correlations are shown by heat map (Figure 4D), where compounds with an asterisk are significantly correlated (**, *p* < 0.01; *, *p* < 0.05). The results showed that under the two treatments, GC, l-aspartic acid, l-threonine, l-glutamine, l-theanine, l-leucine, γ-aminobutyric acid, and l-proline content all showed a significant positive correlation with umami, sourness, and saltiness, while myricetin 3-O-galactoside, vitexin, and l-phenylalanine were significantly negatively correlated with the above three taste factors, which was consistent with the different distribution of the above compounds in CL and SL. The contents of l-threonine, GC, l-theanine, l-glutamine, astragalin, and TF were significantly positively correlated with sweetness. The compounds in tea infusion are complex, and there are interaction relationships. The above correlation results show that these compounds and taste factors have the same trend in the taste contribution of oolong tea infusion.

### 3.3. Dynamic Changes of Free Amino Acid Content in Leaves

**Figure 5:**
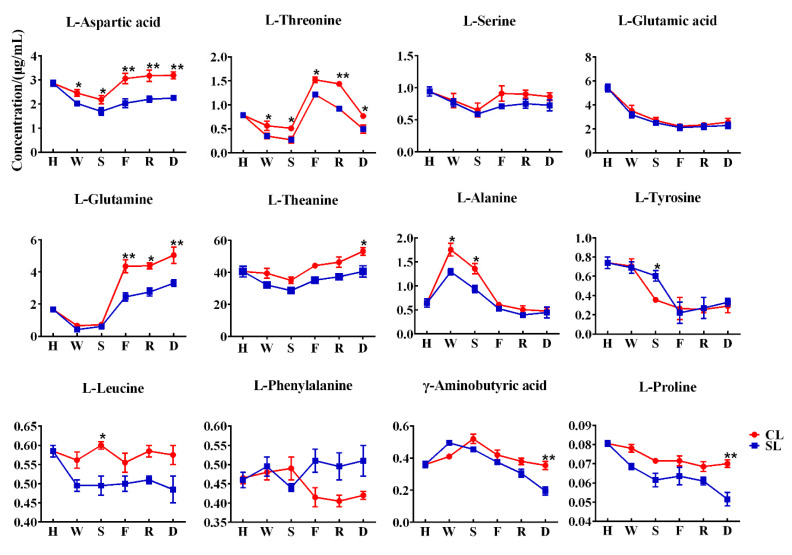
Dynamic changes of free amino acid content in leaves. H stands for harvesting, W stands for withering, S stands for shaking, F stands for firing, R stands for rolling, and D stands for drying (CL, combined leaf; SL, separated leaf; **, *p* < 0.01; *, *p* < 0.05).

The above results show that the existence of stems is conducive to the retention of various free amino acid components related to umami and sweetness in oolong tea. To further explore the effect of stems on the free amino acids of oolong tea during processing, we focused on analyzing the changes of free amino acid components in leaves during processing with stems and processing without stems. The content of free amino acid components in the leaves of the two treatments showed varying trends (Figure 5), indicating that the presence of stems during the processing of oolong tea affected the changes in free amino acid content. We found that the contents of aspartic acid, threonine, alanine, and tyrosine in the two treatments showed significant differences in the early withering and shaking steps. Withering and rotating are the first two processes of oolong tea processing, which mainly affect the taste and aroma of tea through internal biological oxidation [24]. The dynamic changes of enzymatic and nonenzymatic transformations of fresh leaves during processing jointly determine the final sensory perception of oolong tea quality [25,26]. Our results indicated that the existence of stems may affect the biotransformation of free amino acids in tea leaves in the early stage of processing. On the other hand, it can be seen from Figure 4 that the water content of CL was significantly higher than that of SL during the withering and shaking process, indicating that the presence of stems reduced the rate of water loss in leaves, thus possibly slowing the conversion of these free amino acids; however, water redistribution during the slow withering and shaking process may allow stems to transfer these free amino acids from stems to leaves through water transport [12]. The firing process of oolong tea inactivates the enzyme activity, although the subsequent processing does not involve biological reactions, which are mainly related to the formation of tea aroma components [27]. However, these thermodynamically formed aromatic compounds are associated with sugars and amino acids or catechins as substrates [28,29]. The aroma compounds composed of nitrogen-containing compounds may be generated by amino acids donating nitrogen atoms or amino reacting with sugars at high temperatures. Studies have shown that theanine as a substrate during the roasting process contributes to the production of 2,5-dimethylpyrazine, a key compound in the roasting aroma of oolong tea [30]. We found that the glutamine in CL was significantly higher than that in SL after being processed by the firing process, but the differences in alanine and tyrosine were not significant. However, theanine, γ-aminobutyric acid, and proline gradually showed significant differences after the final drying process, indicating that the presence of stems can affect the changes of free amino acids in leaves to varying degrees even under thermal action.

### 3.4. Dynamic Changes of Free Amino Acid Content in Stems

**Figure 6:**
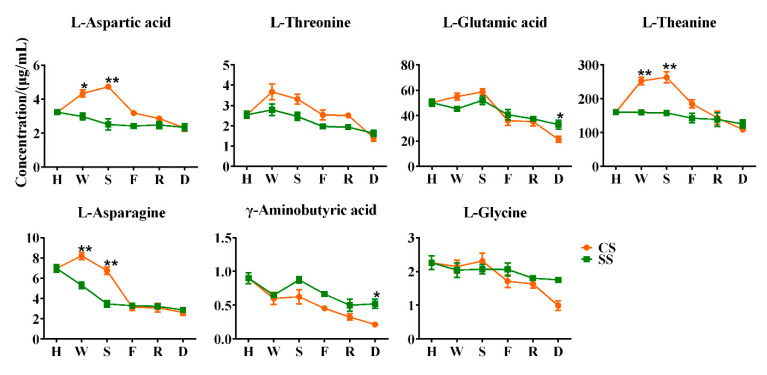
Dynamic changes of free amino acid content in steams. H stands for harvesting, W stands for withering, S stands for shaking, F stands for firing, R stands for rolling, and D stands for drying (CS, combined stem; SS, separated stem; **, *p* < 0.01; *, *p* < 0.05).

Further, we explored whether free amino acids are transported from stems to leaves by moisture during prolonged withering and shaking, as well as the change rule of free amino acid content during processing with only stems. As shown in Figure 6, we determined the content changes of free amino acids in CS and SS during processing. A total of seven free amino acids were detected in SS and CS, of which five were the same as those detected in leaves. We found that in the process of withering and shaking, the free amino acid content in CS was higher than that in SS, except for γ-aminobutyric acid, which was lower in CS than in SS, which is consistent with the corresponding CL and SL, indicating that the marked increase in the content of certain free amino acids during withering and shaking is not related to the transport of water into leaves by stems. In the process after shaking, the content of free amino acids in the stems of CS and SS showed a downward trend, and finally, the content of free amino acids in SS was higher than that in CS, except for aspartic acid. Among them, the difference in glutamic acid and glycine content changed in the process of firing and the differences in the content of threonine, theanine, and asparagine changed in the final drying process, which was all related to the thermal effect at high temperatures.

### 3.5. Comparison of Volatile Components

**Figure 7:**
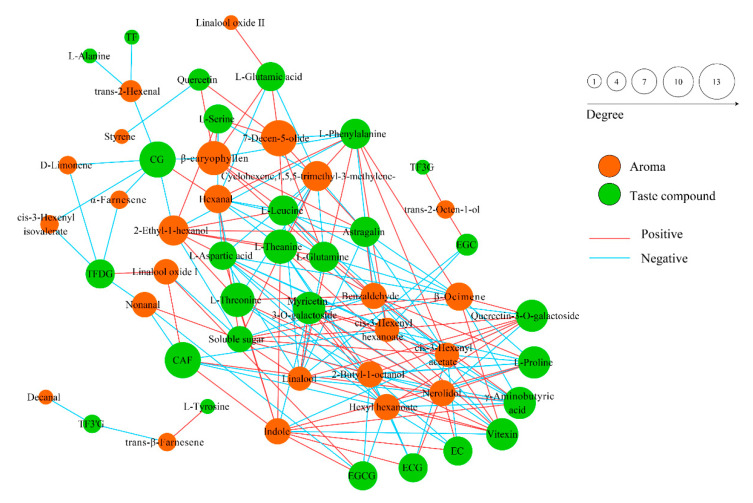
Correlation analysis of aroma components and taste components of two groups of oolong tea samples.

A total of 30 volatile components were detected in finished oolong tea samples, including aldehydes, olefins, alcohols, and their oxides, esters, and nitrogen-containing compounds (Table 1). Aroma molecules in tea are generated from precursors such as carotenoids, lipids, glycosides, and phenylalanine, also from Maillard reactions. Flavonol oxidation is the driving force for carotenoid degradation and participates in the formation of aroma components such as β-ionone and β-damascenone [31]. We conducted a correlation analysis of aroma components and taste components for two groups of oolong tea samples. Through Figure 7, we found that the content of catechins (CG, EC, ECG, EGCG, etc.) was negatively correlated with various aroma components (α-farnesene, Nerolidol, Linalool, etc.). Previous studies have shown that linalool and its oxides, nerolidol, and α-farnesene are the key aroma components in oolong tea and have important contributions to the formation of floral and fruity aromas [32]. On the key aroma components, the nerolidol content of CL was significantly higher than that of SL, while the content of linalool was significantly lower in CL than in SL, and the content of α-farnesene was not significantly different. Phenylalanine is a key precursor of volatile phenylpropanoids/benzenoids (VPBs), which mainly include phenylethyl alcohol, phenylacetaldehyde, benzyl alcohol, benzaldehyde, and coumarin. We see in Figure 7 that phenylalanine was negatively correlated with benzaldehyde content, but benzaldehyde was not significantly different between the two groups.

## 4. Conclusions

This study showed that the participation of stems in the processing of oolong tea changed the overall characteristics of the taste of oolong tea, and the oolong tea with stem processing was significantly higher than the oolong tea without stem processing in terms of umami. In terms of taste components, the content of umami-related free amino acids (such as theanine and aspartic acid) in oolong tea processed with stems was significantly higher than that of oolong teas without stems. However, there were no significant differences in the main catechins, caffeine, and main flavonoids that affect the bitterness of tea, in the theaflavins that affect the astringency of tea, and in the total amount of soluble sugar that affects the sweetness of the tea. In addition, in the biological action of tea leaves under the withering and shaking process and the high-temperature thermal action under the firing and drying process, the stems have different degrees of influence on the content of free amino acids in oolong tea leaves. However, this is not related to the transport of these free amino acids to leaves through the water during the withering and greening process of stems, and the effective mechanism needs to be further studied. In terms of aroma, the nerolidol content of processed oolong tea with stems was significantly higher than that of sessile oolong tea. Although considerably time and expense are required to manually remove the stems at the end of oolong tea processing, the presence of stems during processing is beneficial for the umami of oolong tea. This study provides evidence that helps to elucidate the contribution of stems to the taste quality of oolong tea.

## Figures and Tables

**Table 1:** Content of volatile components in oolong tea.

Retention Time/min	Compound	Content (μg/g) ^a^	
		CL	SL
5.28	Hexanal	4.124 ± 0.127 **	5.494 ± 0.183
7.269	*trans*-2-Hexenal	0.773 ± 0.051	0.839 ± 0.016
8.897	Styrene	0.131 ± 0.022	0.131 ± 0.027
9.553	Heptanal	0.173 ± 0.018	0.180 ± 0.041
12.483	Benzaldehyde	0.184 ± 0.015 **	0.011 ± 0.002
14.157	*trans*-2-Octen-1-ol	0.111 ± 0.013	0.120 ± 0.037
15.564	Octanal	0.086 ± 0.005	0.096 ± 0.004
15.754	*cis*-3-Hexenyl acetate	0.485 ± 0.023 *	0.585 ± 0.038
17.059	d-Limonene	0.189 ± 0.024	0.190 ± 0.036
17.346	2-Ethyl-1-hexanol	0.216 ± 0.039 **	0.135 ± 0.014
17.743	α-Ocimene	0.098 ± 0.007	0.104 ± 0.026
18.416	β-Ocimene	0.215 ± 0.015 *	0.251 ± 0.014
19.925	Linalool oxide II	0.901 ± 0.027	0.797 ± 0.046
20.494	2-Butyl-1-octanol	0.019 ± 0.002 **	0.047 ± 0.001
20.851	Cyclohexene,1,5,5-trimethyl-3-methylene-	0.029 ± 0.005	0.038 ± 0.004
21.019	Linalool oxide I	1.524 ± 0.185	1.325 ± 0.330
22.087	Linalool	13.513 ± 1.290 **	17.257 ± 1.110
22.443	Nonanal	0.591 ± 0.019 *	0.729 ± 0.017
28.507	7-Decen-5-olide	0.392 ± 0.089	0.414 ± 0.064
30.146	Decanal	0.057 ± 0.002	0.050 ± 0.003
30.454	β-cyclocitral	0.063 ± 0.005 *	0.079 ± 0.006
31.736	*cis*-3-Hexenyl isovalerate	0.024 ± 0.003	0.033 ± 0.001
35.301	Indole	5.177 ± 0.281 **	4.121 ± 0.112
40.992	*cis*-3-Hexenyl hexanoate	0.759 ± 0.031 **	0.955 ± 0.030
41.32	Hexyl hexanoate	0.079 ± 0.003 **	0.104 ± 0.023
42.621	β-caryophyllen	0.021 ± 0.002	0.023 ± 0.002
44.812	*trans*-β-Farnesene	0.086 ± 0.003	0.077 ± 0.007
47.459	α-Farnesene	0.216 ± 0.023	0.211 ± 0.002
47.874	δ-Cadinene	0.033 ± 0.001	0.027 ± 0.001
50.211	Nerolidol	2.598 ± 0.173 **	1.523 ± 0.039

Note: a: The aroma content is indicated by mean ± SD, and the significance of the difference is indicated by an asterisk (CL, combined leaf; SL, separated leaf; **, *p* < 0.01; *, *p* < 0.05).

## Data Availability

The data presented in this study are available on request from the corresponding author.
